# Methodological challenges designing pragmatic, multi-centre randomised controlled trials in critical care

**DOI:** 10.1186/1745-6215-14-S1-O125

**Published:** 2013-11-29

**Authors:** Graeme MacLennan, Marion Campbell, John Norrie

**Affiliations:** 1 University of Aberdeen, Aberdeen, UK

## 

Researchers designing pragmatic, multi-centre randomised controlled trials (RCTs) in critical care are faced with numerous potential methodological challenges. These challenges can be outlined using PICO terminology:

## Population

Unlike many other clinical areas, patients admitted to intensive care units (ICUs) do not have easily identifiable diseases (e.g. diabetes or cancer), but are classified as having syndromes with hazy definitions that may be inconsistently applied both within and between centres. The definition and implementation of inclusion and exclusion criteria is therefore difficult, resulting in clinically heterogeneous populations.

## Intervention and control

Due to the rapidly declining condition of patients in ICU “treatment as usual” consists of many therapies that can vary both within and between centres. Often, control and experimental care are complex interventions with minimal differences between treatment protocols and there is a risk of treatment contamination due to creep from control to intervention treatment, particularly when blinding is not feasible. Protocolising control group treatment is essential but non-compliance may be frequent.

## Outcome

The gold standard outcome in critical care research is all-cause mortality reduction. However, ICU patients often have multiple life threatening conditions and RCTs require large sample sizes due to small anticipated effect sizes of interventions on mortality. Alternative outcomes used are composite or combined outcomes. Composite outcomes may be difficult to interpret and combined outcomes, e.g. ventilator free days, pose analysis challenges.

We will discuss the trade-offs between pros and cons of potential solutions to the problems outlined above.

